# BSA-Based Nanoparticles for Dual Loading of Pazopanib and Enzalutamide: Formulation Optimization and In Vitro Evaluation in Breast Cancer Cells

**DOI:** 10.3390/pharmaceutics18040475

**Published:** 2026-04-13

**Authors:** Gizem Ruya Topal, Kubra Kilic, Meral Sarper, Ozgur Esim, Ayhan Savaser, Yalcin Ozkan

**Affiliations:** 1 Department of Pharmaceutical Biotechnology, Gulhane Faculty of Pharmacy, University of Health Sciences, Ankara 06010, Türkiye; gizemruya.topal@sbu.edu.tr (G.R.T.); kubra.kilic@sbu.edu.tr (K.K.); 2 Gulhane Institute of Health Sciences, University of Health Sciences, Ankara 06010, Türkiye; meral.sarper@sbu.edu.tr (M.S.); yalcin.ozkan@sbu.edu.tr (Y.O.); 3 Department of Pharmaceutical Technology, Gulhane Faculty of Pharmacy, University of Health Sciences, Ankara 06010, Türkiye; ayhan.savaser@sbu.edu.tr

**Keywords:** pazopanib, enzalutamide, breast cancer, BSA nanoparticles, 4T1

## Abstract

**Objectives:** Limited intracellular exposure can reduce the in vitro activity of pazopanib (PAZ) and enzalutamide (ENZ). This study developed bovine serum albumin (BSA) particles co-encapsulating PAZ and ENZ (PE-BSA) and evaluated physicochemical properties, release kinetics, 4T1 cellular uptake, and in vitro cytotoxicity versus free drugs and single-drug particles. **Methods:** Drug-loaded BSA particles were prepared using a crosslinking-based method. Particle size (PS), polydispersity index (PDI), zeta potential (ZP), and encapsulation efficiency (EE) were determined. In vitro release was assessed over 48 h and fitted to kinetic models. 4T1 uptake was quantified after 2 and 4 h by intracellular drug levels. Cytotoxicity was measured by MTT at 24 and 72 h (1–100 µg/mL). Moreover, cell death analyses were conducted. Stability studies at +4 °C and serum were also carried out. **Results:** PE-BSA was nanoscale and monodisperse (PS 128.7 ± 2.6 nm; PDI 0.026 ± 0.01) with ZP −31.65 ± 1.13 mV and high EE (PAZ 98.59 ± 1.78%; ENZ 69.79 ± 0.02%). At 24/48 h, cumulative release from PE-BSA was 11.96/12.31% for PAZ and 52.26/85.95% for ENZ. The release kinetics were best described by the Korsmeyer–Peppas model for PAZ (r^2^ = 0.9578) and the Higuchi model for ENZ (r^2^ = 0.9605), indicating diffusion-controlled release. PE-BSA increased 4T1 uptake versus free drugs (2 h: 10.02% PAZ and 21.9% ENZ; 1.77-fold and 4.15-fold), with sustained enhancement at 4 h (2.2- and 4.69-fold, respectively). After 24 h, PE-BSA induced a markedly higher apoptotic response in 4T1 cells (32.5% early apoptosis and 0.8% late apoptosis/early necrosis) compared with free-PAZ (6.6% early apoptosis) and P-BSA (7.3% early apoptosis). Particles were stable. **Conclusions:** PE-BSA produced BSA particles with diffusion-governed release and enhanced 4T1 internalization, supporting albumin particles as a delivery platform to increase intracellular exposure of PAZ/ENZ in vitro.

## 1. Introduction

Breast cancer (BC) is a type of malignancy in which breast cells begin to multiply uncontrollably and replace healthy tissue. BC is the most diagnosed malignancy in women and continues to be a major contributor to cancer-related mortality worldwide. Although current therapeutic options such as surgery, chemotherapy, targeted agents, and immunotherapy have improved patient outcomes, a considerable proportion of individuals still experience poor prognoses and limited long-term survival. The clinical challenge is further intensified by the substantial biological diversity of breast cancer, which comprises multiple molecular and pathological subtypes that differ markedly in behavior, therapeutic response, and overall disease trajectory. This heterogeneity underscores the urgent need for innovative and more effective treatment strategies capable of overcoming existing therapeutic limitations and improving survival rates for patients with this complex disease [1,2,3].

In recent years, the use of new drugs has improved breast cancer treatment across many stages of the disease. Much of this progress has been achieved by combining agents that target different points in the pathways controlling tumor growth and survival. Although these advances have changed the treatment approach, tumor cells can still develop escape mechanisms and resistance. Targeted therapies now offer new opportunities to create effective combinations that block these alternative pathways. Because breast cancer includes many subtypes with distinct signaling routes, designing the right combinations can be difficult. Even so, combination therapy has become standard practice as it can enhance treatment results, allow lower doses, reduce toxicity, and delay the development of resistance [4,5].

The formation of new blood vessels is essential for the initiation, progression, and distant spread of both solid tumors and hematologic cancers. Several growth factors including vascular endothelial growth factor (VEGF), basic fibroblast growth factor (bFGF), and platelet-derived growth factor (PDGF) activate their corresponding receptor tyrosine kinases (RTKs), triggering intracellular signaling pathways that facilitate angiogenic processes. Among these mediators, VEGF is recognized as the principal driver of tumor-associated angiogenesis. Numerous studies have demonstrated that the angiogenic switch constitutes a key limiting step in the overall progression of malignant tumors [6].

Pazopanib (PAZ) is a second-generation tyrosine kinase inhibitor designed for oral administration and can block multiple molecular targets. Its primary activity involves the inhibition of VEGFRs, platelet-derived growth factor receptors, and c-kit signaling pathways that play essential roles in tumor progression and cellular survival. Experimental studies have confirmed that pazopanib effectively suppresses human VEGFR family kinases and several related receptors in vitro. In addition, the compound has produced significant antitumor responses in a range of human xenograft models, notably those representing renal cell carcinoma, as well as breast and lung malignancies [7]. Numerous studies have demonstrated the efficacy of pazopanib in breast cancer [6,8,9]. However, its effectiveness in clinical settings is often constrained due to pronounced adverse effects and the emergence of resistance to treatment.

The androgen receptor (AR) is expressed in nearly 70% of breast tumors, and growing evidence highlights its potential as a therapeutic target in AR-positive breast cancer. Although AR is commonly present in breast malignancies, its functional role appears to be context-dependent, influenced by the surrounding tumor microenvironment as well as the relative circulating levels of estrogens and androgens [10,11]. Enzalutamide (ENZ), a potent AR antagonist that blocks the receptor’s translocation to the nucleus, has been employed in preclinical models of both ER-positive and ER-negative breast cancer to clarify the contribution of AR signaling to tumor behavior [12]. Studies have demonstrated that ENZ has a positive effect on breast cancer [11,12,13].

Recent progress in technology has expanded the use of nanotechnology in medicine, enabling the creation of nanoscale systems for drug delivery. Nanomaterials are especially useful in cancer treatment due to their unique properties, allowing nanoparticles to carry multiple drugs, reach tumors more effectively, and limit side effects. Because of their high loading capacity, lower toxicity, and improved specificity, nanoparticle-based drug delivery has become a promising approach. In breast cancer, these drug-loaded nanoparticles can deliver therapies through passive or active targeting to enhance treatment outcomes [14,15]. A wide range of nanoscale carriers including polymeric nanoparticles, solid lipid nanoparticles, magnetic particles, polymeric micelles, drug-polymer conjugates, nanotubes and dendrimers are currently being developed for different drug delivery applications [16].

Albumin is considered a valuable macromolecular carrier because it is biodegradable, non-toxic, non-immunogenic, easily metabolized into harmless by-products, simple to purify, and highly water-soluble, making it well suited for injectable formulations and nanoparticle production. Albumin-based nanoparticles provide several advantages, including biodegradability, ease of preparation, and good reproducibility. Because many drugs show strong affinity for albumin, this protein matrix can efficiently incorporate a wide range of therapeutic compounds. These nanoparticles typically range from 50 to 300 nm, making them smaller than microparticles, and they often provide more controlled drug release than liposomes, which can enhance patient comfort and treatment adherence. Albumin-based particles can be produced using either bovine serum albumin (BSA) or human serum albumin (HSA) [16,17].

In this study, we aimed to develop BSA-based nanoparticle formulations co-loading PAZ and ENZ to reduce the adverse effects commonly associated with high-dose chemotherapy and to enhance the targeted delivery of these agents through a nanocarrier system. Central Composite Design (CCD) was used to obtain the optimum formulation. The co-encapsulation strategy was designed to strengthen the combined therapeutic effect and achieve synergy in the 4T1 cell line. After preparing the BSA nanoparticles, their physicochemical properties were thoroughly characterized, followed by an evaluation of their cytotoxicity, cellular uptake, and apoptosis-inducing potential in 4T1 cells. This study provides a novel approach for the co-delivery of PAZ and ENZ using a protein-based nanoparticle system, aiming to improve therapeutic efficacy through combination therapy.

## 2. Materials and Methods

### 2.1. Materials

PAZ and ENZ were bought from BLD Pharmatech Ltd. (Shanghai, China). BSA were provided by Thermo Fisher Scientific (Waltham, MA, USA) (The molecular structures of PAZ and ENZ are provided in Supplementary Figure S1). Gluteraldehyde, sodium hydroxide, sodium chloride were bought from Merck (Darmstadt, Germany). (3-(4,5-Dimethylthiazol-2-yl)-2,5-Diphenyltetrazolium Bromide (MTT), DMSO were purchased from Sigma-Aldrich (Hamburg, Germany). Dulbecco’s Modified Eagle’s Medium with high glucose, L-glutamine, sodium pyruvate (DMEM), trypsin-EDTA (0.25%), penicillin–streptomycin, and sterile PBS solution were provided by Vivacell (Denzlingen, Germany). Fetal bovine serum (FBS) was obtained from Serena (Pessin, Germany). The murine mammary carcinoma cell line 4T1 was obtained from the American Type Culture Collection (ATCC, Manassas, VA, USA; CRL-2539). All other chemicals used were of analytical grade.

### 2.2. Preparation of BSA-NPs

BSA nanoparticles were obtained at room temperature using the classical desolvation method with ethanol used as the desolvating agent [18]. First, a weighed amount of BSA was dissolved in distilled water, and the pH of the solution was adjusted with 0.1 M NaOH. PAZ (5 mg) was added to the BSA solution and allowed to incubate for 30 min. Separately, 5 mg of ENZ was dissolved in ethanol. Nanoparticle formation was initiated by adding ethanol to the BSA mixture at a constant rate of 1 mL/min under continuous stirring at 1000 rpm. After 30 min of stirring, the resulting particles were stabilized by crosslinking with an aqueous glutaraldehyde solution (1% (*v*/*v*)), followed by an additional 2 h of mixing. The final nanoparticle suspension was then centrifuged at 18,000 rpm for 30 min using centrifuge (BR-5180-T, Inovia, Buyukcekmece/Istanbul, Türkiye).

### 2.3. Design of BSA Particles

In this study, following preliminary formulation studies, response surface methodology (RSM) was applied to develop BSA nanoparticles and to examine the influence of two independent variables, BSA concentration (*w*/*v*%) (X1) and glutaraldehyde volume (µL) (X2), on particle size (PS) (Y1), polydispersity index (PDI) (Y2), and zeta potential (ZP) (Y3). To evaluate the effects of these factors within the experimental range, a rotatable central composite design (CCD) was employed. The design consisted of factorial, axial, and center points, with an axial distance (α) of 1.414. Due to practical and material constraints associated with nanoparticle preparation, the design included a total of 11 experimental runs with three center points, and was therefore considered an exploratory design rather than a fully replicated CCD. Each independent variable was evaluated at five levels corresponding to coded values (−α, −1, 0, +1, +α). The selected factor ranges were determined based on preliminary formulation studies and the relevant literature to ensure nanoparticle formation and stability within the investigated domain. The experimental combinations were generated using Design Expert 13 software (Stat-Ease, Inc., Minneapolis, MN, USA). The detailed experimental conditions, including both coded and actual values of the independent variables, are presented in Table 1.

### 2.4. In Vitro Characterization Studies

#### 2.4.1. High-Performance Liquid Chromatography (HPLC) Analysis

PAZ and ENZ concentrations were quantified using HPLC system (Agilent 1100, Santa Clara, CA, USA) equipped with a diode-array detector (DAD). Separation was achieved on a C18 column (ACE, Warrington, UK; 4.6 × 250 mm) maintained at 25 °C. For PAZ, the mobile phase consisted of acetonitrile and bidistilled water containing 0.1% (*v*/*v*) o-phosphoric acid (55:45, *v*/*v*). The analysis used a flow rate of 1 mL/min, an injection volume of 10 μL, and detection at 271 nm. The retention time for PAZ was approximately 2.2 min [19].

For ENZ, the mobile phase consisted of acetonitrile and bidistilled water (*v*/*v*) (52:48, *v*/*v*). The analysis used a flow rate of 1 mL/min, an injection volume of 10 μL, and detection at 239 nm. The retention time for PAZ was approximately 3.5 min [20].

#### 2.4.2. Particle Size and Distribution

The particle size (PS) and polydispersity index (PDI) of the nanoparticles were determined by photon correlation spectroscopy (PCS) using a Nicomp Nano Z3000 (PSS, New York, NY, USA). The nanoparticle suspensions were diluted 1:100 with distilled water before measurement. All analyses were performed in triplicate, and results were expressed as the mean z-average size ± standard deviation (SD).

#### 2.4.3. Zeta Potential (ZP)

The zeta potential (ZP) of the nanoparticles was measured using laser Doppler velocimetry (Nicomp Z3000, Entegris, Billerica, MA, USA). The nanoparticle suspensions were diluted 1:50 with distilled water before analysis. Each measurement was performed in triplicate, and the results were reported as mean zeta potential ± standard deviation (SD).

#### 2.4.4. Encapsulation Efficiency (EE%)

The indirect method was used to determine the loaded PAZ and ENZ. After centrifugation, supernatants were collected, and active substances in the supernatant was detected by HPLC (Agilent 1260 Infinity, Waldbronn, Germany). EE% for materials was determined using the following equation:

(1) $$EE\left(\%\right)=\left(\frac{“Total\;drug\;amount-Drug\;amount\;in\;the\;supernatant”}{“Total\;drug\;amount”}\right)\times 100$$

### 2.5. In Vitro Drug Release Study

The release of PAZ and ENZ from the nanoparticles was assessed using the dialysis bag diffusion method at 37 ± 1 °C and 75 rpm under sink conditions. 1 mL of the nanoparticle suspension was placed into a dialysis membrane (MWCO 12–14 kDa, Spectrum Labs, San Francisco, CA, USA) and immersed in 30 mL of PBS (pH 7.4) with %0.1 Tween 80 (*v*/*v*). At predetermined intervals, 1 mL of the medium was withdrawn and replaced with fresh PBS to maintain constant volume. Drug concentrations in the collected samples were quantified by HPLC.

For the free-drug control, the dialysis setup provides an apparent dissolution/diffusion profile across the membrane under the same conditions, whereas kinetic modeling was applied to the nanoparticle-derived release data.

To determine the drug-release mechanism, cumulative release data were fitted to zero-order, first-order, Higuchi, Hixson–Crowell, and Korsmeyer–Peppas models. The model with the highest correlation coefficient (r^2^) was considered the best fit.

### 2.6. Fourier-Transform Infrared (FTIR)Spectroscopy 

FTIR spectroscopy was performed on PAZ, ENZ, BSA, and both the plain and loaded formulations to determine their chemical composition. The spectra were obtained using a Perkin Elmer Spectrum 400 spectrometer (Waltham, MA, USA) across the wavenumber range of 600 to 4000 cm^−1^.

### 2.7. Differential Scanning Calorimetry (DSC)

The thermal behavior of PAZ, ENZ, free-BSA particles, and the PE-BSA formulation was investigated by differential scanning calorimetry (DSC; PerkinElmer, USA). Approximately 1 mg of each sample was compressed in an aluminum pan prior to analysis. Measurements were carried out under a nitrogen atmosphere at a constant heating rate of 10 °C/min over a temperature range of 25–350 °C. The resulting DSC thermograms were analyzed using Pyris software version 9.1.

### 2.8. SEM Characterization

The surface morphology and shape of BSA particles were examined using field-emission scanning electron microscopy (FESEM; FEI Nova Nano SEM 430, Eindhoven, The Netherlands). Freeze-dried samples were coated with a thin gold layer prior to analysis. Imaging was conducted at an accelerating voltage of 20 kV.

### 2.9. Stability Studies for Particles

The formulations were kept at +4 °C for 6 months, and then PS, PDI, and ZP were measured.

#### 2.9.1. Serum Stability

The serum stability of PE-BSA particles was evaluated by incubating the formulations with fetal bovine serum (FBS) at a 1:1 ratio at 37 °C under gentle agitation (50 rpm) for a period of up to 24 h. At predetermined time points, samples were collected, diluted with purified water, and analyzed for PS and PDI using a Nicomp Nano Z3000 analyzer (USA).

### 2.10. In Vitro Cytotoxicity Assay

The cytotoxic effects of free-PAZ, ENZ and loaded BSA particles were evaluated using the MTT assay to determine cell viability. 4T1 cells were cultured in DMEM under standard conditions (humidified atmosphere with 5% CO_2_) and seeded into 96-well plates. Cells were exposed to various concentrations of active substances and nanoparticles for 24 and 72 h, after which cell viability was assessed based on the reduction in the tetrazolium dye. At the end of the exposure period, the wells were washed with PBS and 5 mg/mL MTT solution was added. The cells were then incubated with the MTT reagent for 4 h at 37 °C. Subsequently, the MTT solution was discarded, and the resulting formazan crystals were dissolved in DMSO. Absorbance was recorded at 570 nm using a microplate reader. Untreated cells (medium-only) were used as the negative control, and their viability was considered 100%. Cell viability (%) was calculated using the following equation [21]:

(2) $$Viability(\%)=\left(\frac{Absorbance\;of\;test\;well}{Absorbance\;of\;negative\;control\;well}\right)\times 100$$

### 2.11. In Vitro Cellular Uptake Assay

4T1 cells were maintained as described in the “Determination of Cytotoxicity” section, seeded in 6-well plates at 5 × 10^5^ cells per well, and incubated for 24 h. Fluorescein-labeled BSA nanoparticles were prepared, and free fluorescein was removed by dialysis following labeling. Fluorescein-labeled BSA nanoparticles were added at a concentration of 150 µg/mL, and cells were allowed to interact with the particles for 2 h and 4 h. Cellular uptake was visualized using a fluorescence microscope (Leica, Wetzlar, Germany). Also, for the quantitative determination of PAZ and ENZ uptake by 4T1, following the incubation period, the cells were rinsed two times with ice-cold PBS 7.4, treated with trypsin, and followed by lysing in PBS containing 1% Triton X-100 for 30 min at 37 °C. The solution was analyzed for PAZ and ENZ by a HPLC method described previously. Images of the cells were recorded under a fluorescence microscope (Leica, Wetzlar, Germany).

### 2.12. Cell Death Analysis

Apoptosis in treated 4T1 cells was evaluated using an Annexin-V-FITC/PI staining kit according to the manufacturer’s protocol. Cells were seeded in six-well plates at a density of 5 × 10^5^ and exposed for 24 h to free-PAZ and -ENZ, blank BSA particles, or drug-loaded BSA particles. After treatment, cells were trypsinized, centrifuged, and washed with phosphate buffer, then resuspended in 500 μL of binding buffer. Annexin-V-FITC (5 μL) and PI (5 μL) were added, followed by a 30 min incubation in the dark. Stained cells were subsequently analyzed by flow cytometry (CytoFlex, Beckman Coulter, Brea, CA, USA).

### 2.13. Statistical Analysis

All experiments were performed in triplicate. Data are presented as mean ± standard deviation (SD). Statistical comparisons among multiple groups were performed using one-way analysis of variance (ANOVA) followed by Tukey’s post hoc test for pairwise comparisons. Significance was accepted at *p* < 0.05. Exact *p*-values or ranges are provided in the figure legends where applicable. All analyses were conducted using (GraphPad Prism 9.0, GraphPad Software, San Diego, CA, USA).

## 3. Results

### 3.1. Preparation and Optimization of Particles

**Figure 1:**
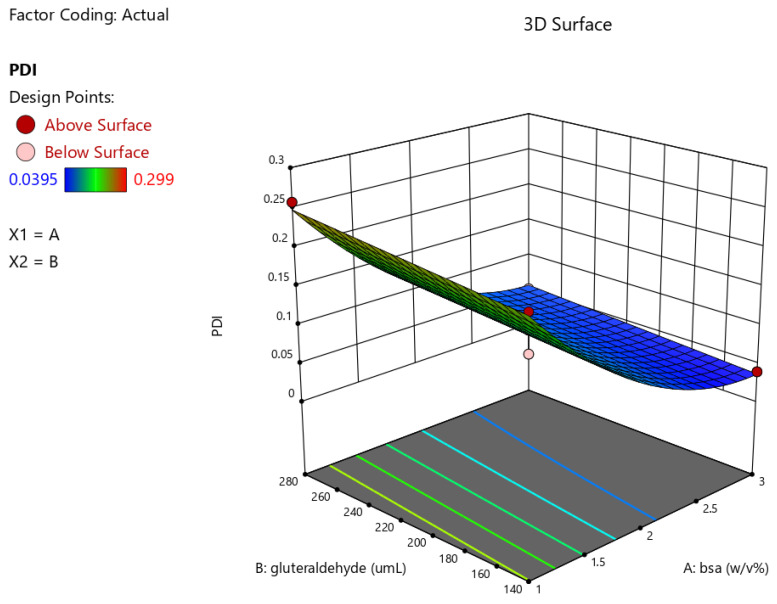
Response surface plot illustrating the effects of formulation variables on PDI derived from response surface methodology (RSM) analysis.

The particles were successfully obtained using the desolvation method. RSM was employed using two factors at three coded levels to assess the effects of BSA concentration (*w*/*v*%) and glutaraldehyde amount on PS, PDI, and ZP. Following the selection of these independent variables, the experiments were carried out based on a CCD, resulting in 11 experimental runs. Experimental design parameters and the measured responses for each run are summarized in Table 2, while the estimated model coefficients are given in Table 3. Graphical representations of the results are provided in Figure 1.

The experimental results obtained from the designed formulations (Table 2) were analyzed using Design Expert software to evaluate the effects of independent variables on the responses. Statistical analysis, including analysis of variance (ANOVA), was performed to determine the significance and suitability of the developed models.

No statistically significant models were obtained for PS and ZP (*p* > 0.05), indicating that these responses were not adequately explained by the selected variables (Table 3 and Table 4).

To further evaluate the adequacy and predictive performance of the developed models, additional statistical parameters were analyzed, as summarized in Table 4.

The obtained data indicated that the nanoparticles exhibited PS ranging from 92.1 to 160.4 nm (Table 2). According to the statistical analysis (Table 3 and Table 4), none of the evaluated models (linear, quadratic, or cubic) for PS were statistically significant (*p* > 0.05), indicating that the selected formulation variables did not adequately explain the variability in PS within the studied design space. Therefore, model-based interpretation of main, interaction, or higher-order effects was not considered appropriate. Instead, PS variations were interpreted descriptively, suggesting that PS was not strongly governed by the investigated factors under the selected experimental conditions. This outcome may be attributed to the influence of additional uncontrolled variables or the limited design resolution. Consequently, PS was not included in the numerical optimization process, and formulation selection was based on the remaining significant responses.

The obtained data indicates that the nanoparticles exhibited a PDI ranging from 0.06 to 0.30 (Table 2). According to Table 3, the quadratic model developed for PDI was statistically significant, as indicated by the model F-value of 33.49 (*p* = 0.0008). The relationship between the independent variables and PDI is described by the following regression equation:

(3) $$Y2\left(PDI\right)=0.0920-0.0921X1+0.0135X2-0.0031X1X2+0.0470{X1}^{2}-0.0000{X2}^{2}$$

Among the model terms, the linear effect of BSA concentration (X1) and its quadratic term (X1^2^) were found to significantly influence PDI, whereas glutaraldehyde amount (X2), the interaction term (X1X2), and X2^2^ were not significant contributors. Overall, the ANOVA results demonstrate that the quadratic model is appropriate for predicting PDI within the investigated design space. The response surface plot illustrating the effect of formulation variables on PDI is presented in Figure 1. As shown in the figure, PDI decreases with increasing BSA concentration at lower levels, followed by a slight increase at higher concentrations, indicating a quadratic behavior. In contrast, glutaraldehyde exhibits a relatively minor influence on PDI within the studied range.

The obtained results showed that the nanoparticles exhibited ZP values ranging from −10.4 to −43.5 mV (Table 2). No statistically significant model was obtained for ZP (*p* > 0.05), indicating that the selected formulation variables did not adequately explain the variability in this response within the studied design space (Table 3). Therefore, model-based interpretation of factor effects was not considered appropriate.

Instead, the results suggest that the zeta potential remained relatively stable across the investigated formulation conditions, possibly due to the dominant influence of intrinsic formulation characteristics rather than the studied variables. The experimental factorial design served as a method to identify the optimal levels of the dependent variables necessary to produce the desired nanoparticles. The objective of this study was to achieve nanoparticles characterized by minimal PS, an optimal ZP, and a low PDI. Therefore, the design was refined to fulfill these specifications [18].

An analysis of the desirability function was conducted in Design Expert to determine the levels of independent variables. Following the optimization study and evaluation of the data presented in Table 2, the formulation suggested by the Design Expert software was prepared and tested. The predicted values generated by the program, along with the experimentally obtained formulation results, are presented in Table 5 [18].

Based on the desirability function analysis of the experimental design, the optimal levels of the independent variables were identified as 2.98% BSA and 158.4 µL glutaraldehyde. The optimization criteria were defined as a PS constrained to 92.05–120 nm, a PDI set to minimize with an upper limit of 0.25, and a ZP targeted within −30 to −20 mV, which yielded a maximum overall desirability (D = 1.000). The predicted response values and the corresponding experimental results are summarized in the relevant tables. However, considering that no statistically significant models were obtained for PS and ZP, the optimization results were interpreted cautiously. Using these suggested levels, the model-guided formulation produced nanoparticles with a mean size of 115 nm, a PDI of 0.028, and a zeta potential of −21.8 mV.

After evaluating the experimental data for the optimized formulation containing 2.98% BSA concentration and 158.4 µL glutaraldehyde volume in terms of PS, PDI, and ZP as presented in Table 5, the encapsulation efficiency was subsequently assessed. The formulations were reprepared in both single and combined ones. Table 6 presents a summary of the experimental results obtained from both single and combined formulations. The size distribution curves for all formulations are provided in the Supplementary Information (Figure S2).

### 3.2. Results of In Vitro Drug Release Study

**Figure 2:**
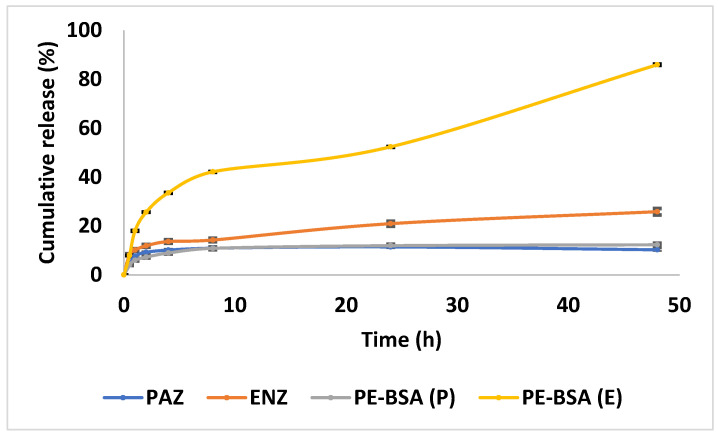
In vitro cumulative release profiles of PAZ and ENZ from the PE-BSA formulation compared with free-PAZ and free-ENZ controls. Data are presented as mean ± SD (*n* = 3) over a 50 h release period. Error bars are not visible due to the very small standard deviation values.

The cumulative release profiles of free-PAZ, ENZ and PE-BSA nanoparticles are presented in Figure 2.

During the first 24 h, the cumulative release of free-PAZ and free-ENZ was 11.45% and 20.93%, respectively. In contrast, the combined formulation (PE-BSA) exhibited a comparable release profile for PAZ (11.96%), whereas the release of ENZ was notably higher, reaching 52.26% over the same period.

At the end of 48 h, the cumulative release was 11.50% for free-PAZ and 25.82% for free-ENZ. For the PE-BSA formulation, the release of PAZ reached 12.31%, while ENZ release increased to 85.95%. By the end of the 48 h period, the release profile of PAZ remained essentially unchanged, whereas ENZ exhibited a further increase in release into the medium.

To clarify the release behavior, the cumulative release profiles were analyzed using various kinetic models. Following correction of the time-point inconsistencies, the kinetic analysis was repeated. As shown in Table 7, the release profile of PAZ was best described by the Korsmeyer–Peppas model (r^2^ = 0.9578), indicating a diffusion-controlled release mechanism, as further supported by the release exponent value (*n* = 0.3757), which is characteristic of Fickian diffusion. In contrast, the release of ENZ showed the highest correlation with the Higuchi model (r^2^ = 0.9605), although other models also demonstrated relatively good fits. This suggests that ENZ release is primarily governed by diffusion, with possible minor contributions from additional mechanisms. Overall, these findings indicate that drug release from the PE-BSA formulation is predominantly diffusion-driven, with slight differences between the two active compounds. The corresponding fitting plots are provided in the Supplementary Information (Figure S2).

### 3.3. FTIR Spectral Analysis

**Figure 3:**
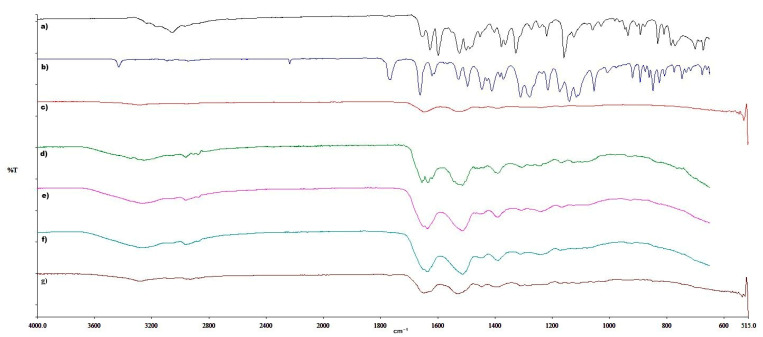
FTIR spectrums of (a) PAZ; (b) ENZ; (c) BSA; (d) free-BSA; (e) P-BSA; (f) E-BSA; (g) PE-BSA.

FTIR spectra of PAZ, ENZ, BSA, plain, and loaded BSA particles were recorded on a Spectrum 400 IR-spectrophotometer (Figure 3). The FTIR spectrum of PAZ exhibited characteristic absorption bands corresponding to C–H stretching vibrations at 3056 cm^−1^, C=C stretching at 1655 cm^−1^, N–H bending at 1500 cm^−1^, S=O stretching of the sulfonamide group at 1377 cm^−1^, and aromatic C–N stretching at 1327 cm^−1^. These spectral features are consistent with those reported in the literature [22,23].

The absorption band at 3430 cm^−1^ corresponds to the N–H stretching vibration of the amide (-CONH-) group in enzalutamide, while the peak observed at 1764 cm^−1^ is attributed to C=O stretching vibrations. These spectral features are consistent with those reported in the literature [24,25].

In the spectra of plain BSA particles, characteristic amide I (1650 cm^−1^) and amide II (1540 cm^−1^) bands were observed, confirming the protein structure. For PAZ and ENZ loaded BSA particles, the characteristic peaks of the free drugs were no longer visible. Moreover, minor shifts in the amide I and II bands of BSA were detected, suggesting possible hydrogen bonding or other non-covalent interactions between the drugs and the protein matrix.

### 3.4. DSC Thermogram Analysis

**Figure 4:**
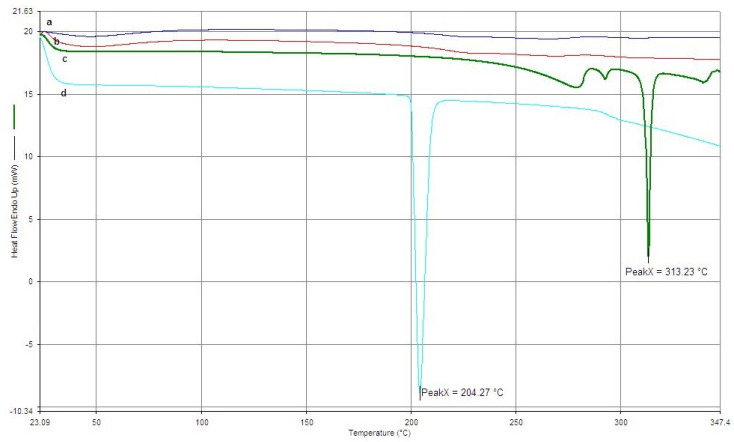
DSC thermograms of (a) PE-BSA; (b) free-BSA; (c) PAZ; (d) ENZ.

DSC was utilized to analyze the thermal properties of the materials that were used for the formulation and nanoparticles. When the DSC thermograms of PAZ, ENZ and formulations were examined, the endothermal peaks were seen at 313.3 °C, 204.27 °C for PAZ and ENZ respectively [24,26]. The thermograms of the formulations did not exhibit the characteristic melting peaks of the active compounds (Figure 4). However, no distinct thermal peaks were observed in the thermograms of the formulations. The disappearance of the characteristic peaks associated with the active compounds suggests that the drugs were successfully encapsulated within the nanoparticle matrix.

### 3.5. SEM Analysis

**Figure 5:**
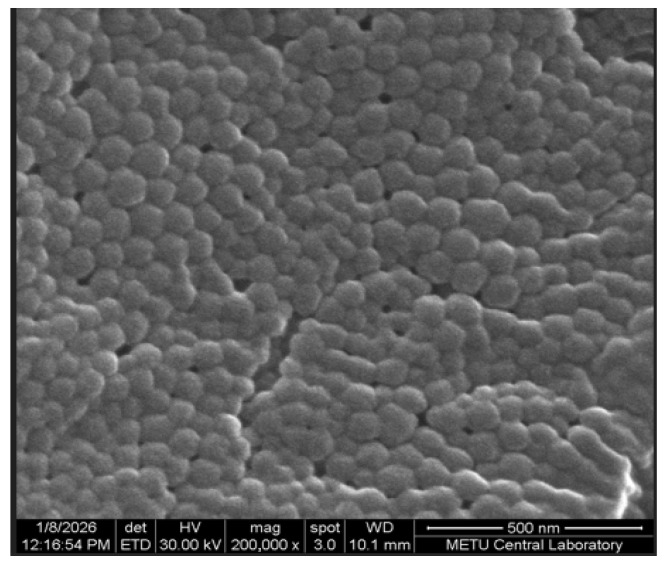
SEM images of particles.

The morphological features of BSA particles were evaluated by FE-SEM. As shown in Figure 5, the nanoparticles exhibited a spherical geometry and a homogeneous size distribution.

### 3.6. Stability Studies for SLNs

The physical stability of the PE-BSA formulations was evaluated by tracking changes in PS, PDI and ZP following storage at 4 °C for six months. As summarized in Table 8, the formulations exhibited a mild increase in PS and PDI over time, indicating limited particle aggregation during storage. In contrast, ZP values showed a significant reduction (*p* < 0.05), suggesting a decrease in surface charge stability.

Upon evaluation of serum stability, a slight increase in particle size was observed, whereas a reduction in PDI was detected. The corresponding results are presented in Table 9.

### 3.7. Cell Culture Studies

#### 3.7.1. Determination of Cytotoxicity

**Figure 6:**
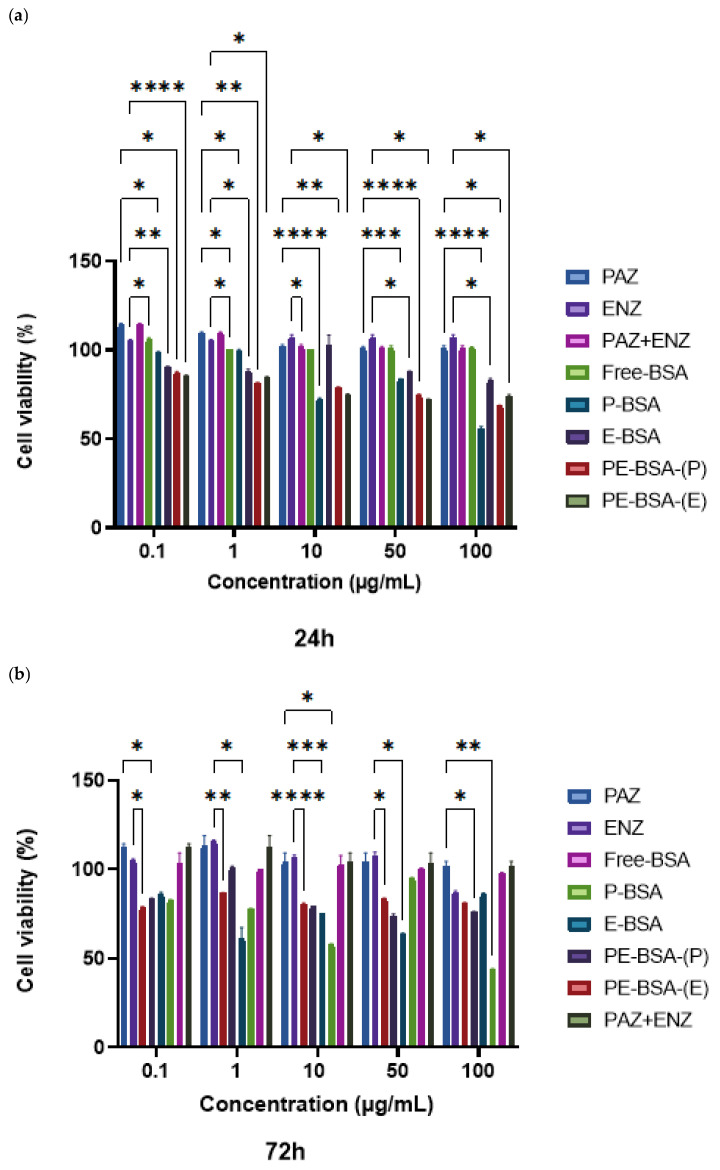
MTT cell viability results in 4T1 cells after exposure to free drugs and BSA particle formulations for (**a**) 24 h and (**b**) 72 h. Data are presented as mean ± SD (*n* = 3). Statistical significance was determined by one-way ANOVA followed by Tukey’s post hoc test. Only statistically significant differences are indicated (* *p* < 0.05, ** *p* < 0.01, *** *p* < 0.001, **** *p* < 0.0001).

Dose/response data indicated that free-BSA particles preserved high levels of cell viability throughout the tested range, confirming minimal carrier associated toxicity. In comparison, incorporation of drugs into the nanoparticles resulted in clearly altered cytotoxicity patterns. Both P-BSA and E-BSA yielded the greatest loss of viability at 100 µg/mL at 24 and 72 h. When the co-loaded system PE-BSA was evaluated against the single-loaded formulations, it showed stronger cytotoxicity than E-BSA, whereas no further increase was observed relative to P-BSA, implying that co-encapsulation preferentially improved ENZ-related activity. Collectively, the nanoformulations-especially PE-BSA-demonstrated substantially enhanced efficacy versus the corresponding free-drug treatments, highlighting the utility of BSA particle mediated encapsulation (Figure 6).

#### 3.7.2. Cellular Uptake Results

**Figure 7:**
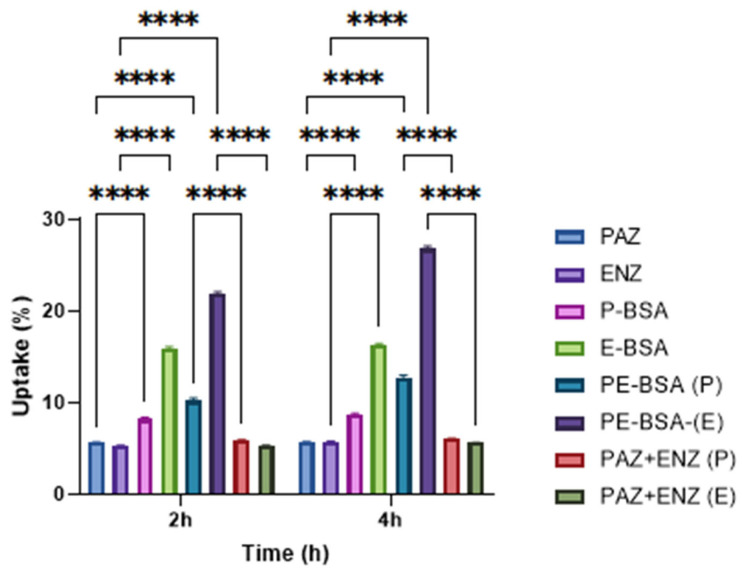
Cellular uptake results of free active substances and particles. Data are presented as mean ± SD (*n* = 3). Statistical significance was determined by one-way ANOVA followed by Tukey’s post hoc test. Only statistically significant differences are indicated (**** *p* < 0.0001). (PAZ + ENZ represents the combination of free-PAZ and free-ENZ).

The cellular uptake of PAZ and ENZ by 4T1 cells is shown in Figure 7. After 2 h of incubation, intracellular drug levels were 5.65% and 5.27% for free-PAZ and free-ENZ, respectively, while PAZ uptake from P-BSA was 8.27%. In the combined free-drug treatment, uptake values were 5.93% for PAZ and 5.33% for ENZ, indicating no substantial enhancement compared with the single-drug treatments. In contrast, markedly higher uptake was observed for the PE-BSA formulation, reaching 10.02% (PAZ) and 21.9% (ENZ) after 2 h. This corresponds to a 1.77-fold increase relative to free-PAZ and a 4.15-fold increase relative to free-ENZ (*p* < 0.0001). Notably, PAZ uptake from PE-BSA was approximately 1.2-fold higher than that from P-BSA at the same incubation time.

After 4 h of incubation, uptake from PE-BSA further increased, remaining approximately 2.2-fold higher than free-PAZ and 4.69-fold higher than free-ENZ (*p* < 0.0001). Overall, these findings demonstrate that PE-BSA particles are efficiently internalized by 4T1 cells and can increase intracellular exposure compared with free drugs and the single-drug formulation.

**Figure 8:**
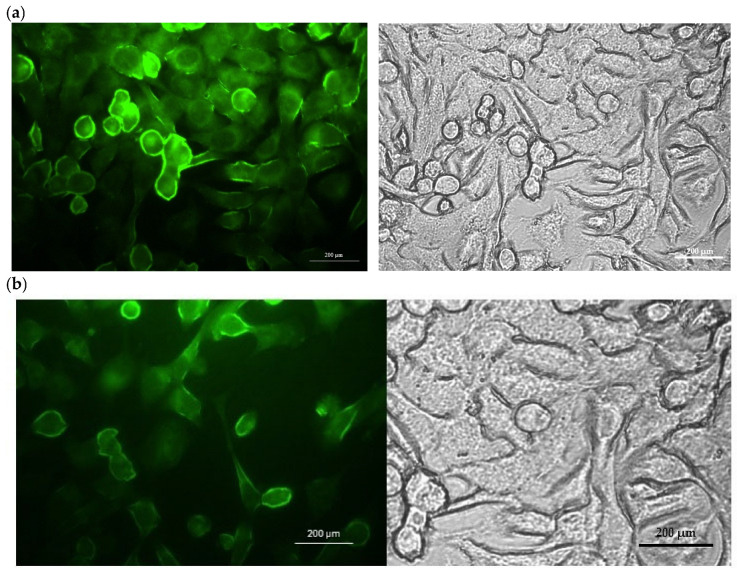
Representative cellular uptake microscopy images of (**a**) free-BSA particles and (**b**) dye solution in 4T1 cells. Fluorescence and corresponding brightfield images are shown to confirm cell morphology and signal localization.

Moreover, when Figure 8 is examined, it is clearly observed that particles enter the cell. It is clearly observed from Figure 8 that the particles (Figure 8a) are internalized into the cells more than the free dye (Figure 8b).

#### 3.7.3. Cell Death Analyses

**Figure 9:**
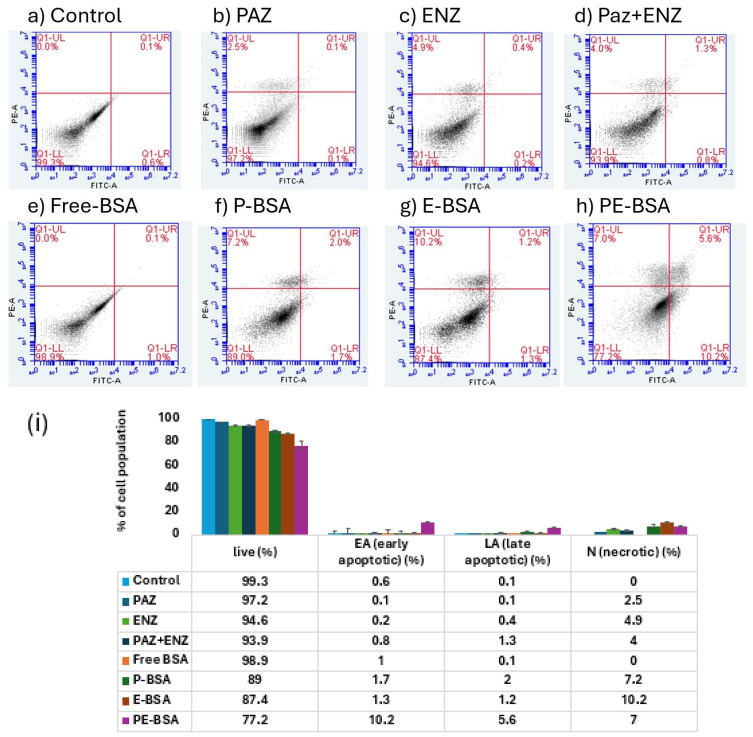
(**a**–**h**) Flow cytometry results for PAZ, ENZ and particles; (**i**) Apoptosis results.

The apoptotic activity of free PAZ, free-ENZ, free-Paz + ENZ, free-BSA particles, P-BSA, E-BSA and PE-BSA formulations in 4T1 cells was analyzed by flow cytometry using Annexin-V-FITC and propidium iodide (PI) dual staining (Figure 9).

Evaluation of the apoptosis data revealed clear differences among the treatment groups after 24 h. Cells treated with free-PAZ exhibited a limited apoptotic response with 2.5% necrotic (N) (Annexin-V-FITC^+^/PI^+^) cells, while no significant early or late apoptotic populations (EA or LA) were detected. Similarly, cells treated with free-ENZ showed only a minor apoptotic response (0.2% EA, 0.4% LA and 4.9% N). When the free-PAZ + ENZ combination was applied, a slightly increased apoptotic population was observed compared with the individual free-drug treatments (0.8% EA, 1.3% LA and 4.0% N).

In comparison, free-BSA particle-treated cells exhibited low apoptotic levels (1.0% EA) and 0.1%LA), confirming the minimal cytotoxicity associated with the carrier system. Treatment with the single-drug nanoparticle formulations resulted in moderately increased apoptosis. Specifically, P-BSA treatment produced 1.7% EA and 2.0% LA cells, while E-BSA treatment also induced a modest increase in apoptotic cells compared with the corresponding free-drug group (1.3% EA, 1.2% LA and 10.2% N).

Notably, cells treated with the co-loaded PE-BSA formulation exhibited the most pronounced apoptotic response, characterized by 10.2% early apoptotic cells and 5.6% late apoptotic cells. Overall, while both free-drug combinations and single-drug nanoparticle formulations induced measurable apoptotic responses, the PE-BSA formulation produced the highest level of apoptosis, indicating that the co-delivery of PAZ and ENZ within BSA nanoparticles markedly enhances apoptosis induction in 4T1 cells. These findings are illustrated in Figure 9.

## 4. Discussion

Breast cancer is the most frequently diagnosed malignancy worldwide and remains a leading cause of cancer-related mortality among women. In therapy, the use of multiple therapeutic agents has become increasingly important, as targeting distinct biological pathways can improve treatment outcomes and help limit the adverse effects typically seen with escalated doses of single chemotherapeutic drugs. In the present study, PAZ and ENZ were co-encapsulated into BSA nanoparticles, and the resulting formulations were prepared and characterized. The anticancer effects of these co-loaded nanoparticles were subsequently assessed in the 4T1 cell line.

The desolvation method is one of the most commonly used methods for obtaining BSA particles and has been successfully applied in our study. Key factors for the successful formulation development include an optimal PS, suitable ZP, and a narrow, uniform size distribution. Because of this reason, BSA particles were examined in terms of PS, PDI and ZP. RSM was applied to obtain the optimum formula. The primary benefit of RSM is that it minimizes the amount of experimental work required while allowing each influencing factor to be evaluated individually to identify optimal conditions. The central composite design (CCD) is well suited for stepwise experimental studies, as it provides sufficient information to evaluate lack of fit while requiring a relatively small number of experimental runs [27].

Particle size played a key role during formulation optimization, as smaller particles provide a larger surface area, which in turn promotes improved drug solubility [28]. The concentration of BSA has been shown to directly impact nanoparticle size, with higher concentrations often leading to larger particles in the literature [29]. According to our results, PS is mainly influenced by BSA concentration (X1). An increase in BSA concentration was associated with a corresponding increase in PS, a trend that is consistent with previously reported findings in the literature. Increasing BSA concentration promoted particle coagulation and resulted in larger particle sizes. This behavior is likely related to the higher solution viscosity, which slows protein diffusion between the aqueous and ethanol phases, thereby reducing nucleation and favoring the formation of larger particles [29,30]. In contrast, the effect of glutaraldehyde amount on PS is relatively weak when considered alone. When the results of the studies are examined, the data obtained supports our findings [31]. Nevertheless, when glutaraldehyde is combined with BSA, especially at higher crosslinker levels, their interaction becomes more pronounced and can contribute to an increase in PS. Overall, these findings demonstrate that PS is primarily affected by BSA concentration and its interaction with glutaraldehyde, while the crosslinker amount by itself plays a less dominant role.

PDI is used to describe the width of particle size distribution. Colloidal systems with PDI values below 0.2 are generally regarded as having a uniform and well-dispersed particle population [32]. According to the regression equation, BSA concentration (X1) is the dominant factor influencing PDI, exhibiting a nonlinear effect in which moderate levels result in lower PDI values. In contrast, glutaraldehyde amount (X2) shows only a minor contribution to PDI, with negligible quadratic and interaction effects. These findings indicate that PDI is primarily governed by BSA concentration rather than crosslinker amount [31,33].

Zeta potential reflects the surface charge of nanoparticles and plays a crucial role in maintaining the physical stability of colloidal dispersions by promoting electrostatic repulsion between particles. In general, zeta potential values approaching ± 30 mV are considered indicative of highly stable nanoparticle systems. The negative surface charge of nanoparticles is attributed to the negative charge of BSA [34]. The statistical analysis indicated that the model was ineffective in predicting the ZP response.

The formulation recommended by the design program was prepared using 2.98% BSA and 158.4 µL of glutaraldehyde solution. The experimentally obtained PS, PDI, and ZP values were found to be in close agreement with the values suggested by the program. However, considering that no statistically significant models were obtained for PS and ZP, these results were interpreted cautiously. Following the selection of this optimum formulation, PAZ and ENZ were loaded individually as well as in combination, and the EE% was subsequently calculated. Despite the very low aqueous solubility of PAZ and ENZ, high encapsulation efficiency values (P-BSA: 97.82%; E-BSA: 70.53%; PE-BSA (PAZ): 98.59%; PE-BSA (ENZ):69.79%) were obtained. This phenomenon is not unique to the current study, as previous research has also highlighted BSA’s efficacy in improving the solubility and entrapment efficiency of other poorly water-soluble drugs [35,36]. This outcome is thought to be attributable to the solubility-enhancing effect of bovine serum albumin, as also reported in the literature [37].

The lower EE of ENZ compared with PAZ likely reflects drug-dependent partitioning and drug–albumin interactions during particle formation. Albumin contains hydrophobic binding regions and multiple polar interaction sites; compounds with stronger affinity and retention within the albumin-rich phase may be entrapped more efficiently and further stabilized upon crosslinking. In contrast, compounds that remain more weakly associated or display greater partitioning into the external medium can be partially lost during purification, leading to a lower apparent EE [30,38].

The markedly higher release of ENZ from the PE-BSA formulation, despite its poor aqueous solubility, may arise from differences in drug–albumin interactions and the intraparticle distribution of the drug within the crosslinked BSA matrix. ENZ may be present to a greater extent as a loosely bound or surface-associated fraction and could be more readily mobilized under the release conditions, whereas PAZ appears to be more strongly retained within the matrix, resulting in a limited and plateauing release profile [39]. Albumin can form reversible complexes with hydrophobic drugs, increasing their apparent solubility and facilitating diffusion into the release medium. However, the magnitude of this solubilization effect depends on the binding strength and dissociation kinetics of the drug–albumin interaction. Accordingly, compounds that are more weakly bound or more readily dissociate from albumin may display enhanced apparent release, while more strongly retained drugs may remain associated with the albumin matrix and exhibit slower or plateauing release [40,41].

The release behavior of the BSA nanoparticles was evaluated using different kinetic models, revealing distinct patterns for the two drugs. While ENZ release was best described by the Higuchi model (r^2^ = 0.9605), PAZ release showed the highest correlation with the Korsmeyer–Peppas model (r^2^ = 0.9578).

The good fit of ENZ to the Higuchi model supports a diffusion-driven release mechanism, consistent with the assumption of drug diffusion from a relatively stable matrix where structural changes such as swelling or erosion are minimal. In this context, the crosslinked BSA nanoparticle matrix likely serves as a stable carrier system, enabling gradual diffusion of ENZ through the protein network.

For PAZ, the Korsmeyer–Peppas model provided the best fit, with a release exponent (*n* = 0.3757) indicative of Fickian diffusion. This further confirms that diffusion is the dominant release mechanism, although the difference in the best-fit model compared to ENZ suggests that drug-specific properties, such as molecular interactions with the BSA matrix or solubility characteristics, may influence the release kinetics. Overall, these findings indicate that drug release from the developed BSA-based nanoparticles is predominantly governed by diffusion processes, with slight variations depending on the physicochemical properties of the encapsulated drug, rather than by matrix degradation or significant structural changes during the release period [39].

FTIR analysis confirmed the incorporation of PAZ and ENZ into the BSA particle matrix. The absence of the characteristic PAZ and ENZ peaks in the loaded particle spectra indicates that the drugs were effectively encapsulated and uniformly distributed. The slight shifts observed in the amide I and II bands of BSA further suggest potential hydrogen bonding or electrostatic interactions between the protein and the incorporated drugs, which may contribute to particle stability and controlled release. These results demonstrate successful drug entrapment without significant alteration of the BSA secondary structure.

DSC analysis confirmed successful incorporation of PAZ and ENZ into the BSA nanoparticle matrix. The absence of the characteristic drug melting peaks in the loaded particle thermograms demonstrates effective encapsulation. Slight shifts in the BSA denaturation peak indicate possible hydrogen bonding or other interactions between the protein and the incorporated drugs, which may contribute to particle stability. Overall, the thermal analysis supports that drug entrapment occurred without major disruption of the BSA matrix.

SEM provides high-resolution images of the surface morphology of BSA nanoparticles. Upon examination of the SEM images, it was clearly seen that particles with a homogeneous distribution and spherical shape have been obtained. When looking at the PS, it was seen that they were smaller than those measured by DLS. This situation was thought to be due to the albumin swelling slightly in the aqueous environment. The particles may have shrunk slightly due to the absence of water in the environment after lyophilisation. This finding is consistent with the literature data [40].

Particle size is a key determinant of nanoparticle performance in systemic circulation, influencing interactions with cells, circulation time, target recognition, and cellular uptake. Accordingly, drug delivery systems are ideally designed to achieve the smallest possible particle size while maintaining sufficient stability, as stable nanoscale carriers are more likely to exhibit favorable pharmacokinetic and targeting characteristics [28]. Evaluation of the stability results further demonstrated that the nanoparticles retained an appropriate size range over a six-month storage period. Regarding long-term stability, the zeta potential of the PE-BSA nanoparticles decreased from −31.65 mV to −4.67 mV over six months at 4 °C. This reduction likely results from minor protein rearrangement, adsorption of ions from the storage medium, or slight aggregation, which are common phenomena in protein-based colloidal systems. Despite this decrease, the nanoparticles maintained their overall size distribution without visible precipitation, indicating that functional colloidal stability was largely preserved during storage. This observation is supported by previous studies reporting that albumin-based nanoparticles show a decrease in zeta potential over storage, associated with minor aggregation and surface interactions, without complete loss of colloidal stability [42]. Overall, these results indicate that while minor variations in surface charge and zeta potential can occur over time, the PE-BSA formulation remains physically stable and suitable for further experimental applications.

The serum stability of the nanoparticles was evaluated with regard to their proposed intravenous application, to assess potential particle aggregation in a protein-rich environment. Upon incubation with fetal bovine serum, the particle size increased slightly from 144.6 ± 0.35 nm to 151.3 ± 0.074 nm, likely due to adsorption of serum proteins and other biomolecules onto the particle surface [41]. Despite this increase, the PS remained below 200 nm, which is considered critical for effective interaction with cancer cells; nanoparticles smaller than 150 nm exhibit superior cellular uptake, correlating with enhanced cytotoxicity [43,44]. Interestingly, the PDI decreased from 0.15 ± 0.02 to 0.07 ± 0.0014. This decrease does not indicate aggregation; rather, it reflects a more uniform particle population, likely influenced by serum-induced surface stabilization and the intensity-weighted nature of DLS measurements, where minor subpopulations contribute less to the reported distribution [45]. Thus, the nanoparticles maintain a stable and monodisperse population in the serum environment.

MTT results indicated that, for ENZ, particle loading and subsequent administration in the combined formulation led to increased cytotoxicity at both 24 and 72 h. In contrast, for PAZ, P-BSA increased cell death compared with the free-PAZ; however, no additional increase in cytotoxicity was observed under the combined treatment condition. The release data of the co-loaded formulation revealed different profile, with ENZ being liberated faster from PE-BSA while PAZ exhibited a slower release. This kinetic difference may explain why PE-BSA did not further increase PAZ-related cytotoxicity compared with P-BSA within the 24–72 h window, as the bioavailable PAZ fraction could remain limited. In contrast, the faster ENZ release from PE-BSA may enhance early exposure and contribute to the higher cytotoxicity observed relative to E-BSA [46].

The cellular uptake results indicate that PE-BSA particles were efficiently internalized by 4T1 cells. BSA nanoparticles are widely considered biocompatible and biodegradable carriers, and their surface characteristics can be tuned to improve cellular internalization and intracellular drug delivery compared with free drugs [47]. In cancer cells, uptake of albumin carriers may be facilitated by albumin-associated pathways, including interactions with albumin-binding proteins/receptors that have been reported to contribute to the cellular handling and accumulation of albumin-based systems [40,48]. Notably, the co-loaded formulation (PE-BSA) resulted in approximately 1.2-fold higher cellular uptake of PAZ compared with the PAZ-only formulation (P-BSA). This increase is reported as an observation without speculating on the direct effects of ENZ. Consistent with previous reports, co-delivery within albumin carriers can enhance overall nanoparticle internalization and drug accumulation relative to single-drug loading, potentially by altering physicochemical properties and leveraging albumin-associated uptake routes [49].

The apoptotic activity of free active substances and particles in 4T1 cells was evaluated by flow cytometry using Annexin V–FITC/PI dual staining. Apoptosis is a tightly regulated process that contributes to cellular homeostasis and represents a key mechanism exploited by anticancer therapies. An early hallmark of apoptosis is phosphatidylserine (PS) externalization from the inner to the outer leaflet of the plasma membrane; Annexin V–FITC binds exposed PS and thereby identifies early apoptotic cells. At later stages, loss of membrane integrity permits PI entry, resulting in Annexin V/PI double-positive populations, while PI positivity may also indicate necrosis due to severe membrane disruption [50,51]. The apoptosis analysis provided further insight into the cytotoxic mechanisms of the developed formulations. Cells treated with the free-drug forms exhibited limited apoptotic responses, indicating that the intrinsic activity of the drugs alone induced only modest apoptosis under the tested conditions. Although the free-PAZ + ENZ combination slightly increased apoptotic cell populations compared with the individual drugs, the effect remained moderate, possibly due to limited intracellular availability of the free drugs. In contrast, BSA-based nanoparticle formulations enhanced apoptosis induction. The single-drug nanoparticles (P-BSA and E-BSA) produced higher apoptotic levels than their corresponding free drugs, likely due to improved cellular uptake and intracellular delivery mediated by the nanoparticle carrier. Among all groups, the co-loaded PE-BSA formulation generated the most pronounced apoptotic response, suggesting that the simultaneous intracellular delivery of both drugs more effectively activates apoptotic pathways. Overall, these findings demonstrate that nanoparticle-mediated delivery enhances drug-induced apoptosis, while co-encapsulation of PAZ and ENZ in BSA nanoparticles further amplifies this effect. These results also support the utility of nanoparticle-enabled co-delivery strategies to enhance intracellular drug exposure and biological activity compared with single-drug treatments, highlighting the therapeutic potential of the combined nanoparticle system [52].

## 5. Conclusions

In conclusion, PAZ and ENZ loaded BSA particles (PE-BSA) were successfully developed with nanoscale size, low dispersity, and high encapsulation efficiency, indicating robust formulation performance. The co-loaded PE-BSA system displayed diffusion related release kinetics and markedly enhanced cellular internalization in 4T1 cells compared with free drugs. Although cytotoxicity profiles differed between single and combined drug formulations, PE-BSA produced measurable reductions in cell viability and elicited a pronounced apoptotic response, supporting improved intracellular pharmacological activity upon co-encapsulation. Overall, these findings demonstrate that BSA-based particles are a feasible delivery platform for PAZ-ENZ and warrant further mechanistic validation and evaluation in disease-relevant models to clarify the determinants of efficacy and optimize the drug ratio and exposure conditions.

## Figures and Tables

**Table 1:** Experimental design and characteristics of nanoparticles.

Variables	Levels		
	Low (−1)	Medium (0)	High (+1)
X1: BSA concentration (*w*/*v*%)	1	2	3
X2: Glutaraldehyde volume (µL)	140	210	280
**Dependent Variables**			
Y1: Particle size (nm)	Minimize		
Y2: Polydispersity index Y3: Zeta potential (mV)	In range +30/−30 mV		

**Table 2:** Formulation parameters and the measured responses for each particle.

Formulation Code	Independent Variables		Responses		
	X1: BSA Concentration (*w*/*v*%)	X2: Glutaraldehyde Volume (µL)	PS (nm ± SD)	PDI (Mean ± SD)	ZP (mV ± SD)
F1	2	111.005	116.0 ± 4.5	0.06 ± 0.06	−19.8 ± 0.9
F2	1	280	112.0 ± 1.1	0.26 ± 0.02	−14.4 ± 2.6
F3	3	140	115.8 ± 2.9	0.04 ± 0.01	−28.8 ± 4.1
F4	2	210	101.3 ± 1.9	0.09 ± 0.03	−13.5 ± 1.3
F5	1	140	92.1 ± 0.3	0.23 ± 0.01	−39.4 ± 0.2
F6	3	280	121.8 ± 2.7	0.05 ± 0.00	−40.9 ± 3.7
F7	3.41421	210	122.3 ± 2.6	0.06 ± 0.04	−43.5 ± 4.2
F8	0.585786	210	160.4 ± 2.4	0.30 ± 0.06	−21.8 ± 2.0
F9	2	210	107.2 ± 1.0	0.06 ± 0.00	−29.4 ± 2.4
F10	2	308.995	113.2 ± 1.9	0.11 ± 0.04	−10.4 ± 5.5
F11	2	210	119.5 ± 3.6	0.12 ± 0.08	−31.1 ± 0.9

Note: Data were represented as mean ± SD (*n* = 3).

**Table 3:** Summary of the response parameters.

Source	Sum of Squares	df	Mean Square	F	*p*-Value
***PS (No significant model (p > 0.05))***					
**X1**	727.71	1	727.71	2.77	0.1947
**X2**	3.92	1	3.92	0.0149	0.9105
**X1X2**	49.35	1	49.35	0.1878	0.6940
**X1^2^**	764.24	1	764.24	2.91	0.1867
**X2^2^**	16.88	1	16.88	0.0642	0.8163
**X1^2^X2**	111.82	1	111.82	0.4255	0.5607
**X1X2^2^**	954.89	1	954.89	3.63	0.1527
**X1^3^**	0.0000	0	0.0000		
**X2^3^**	0.0000	0	0.0000		
***PDI (Model: Quadratic;*** **r^2^: 0.9710*)***					
**X1**	0.0678	1	0.0678	136.90	˂0.0001
**X2**	0.0015	1	0.0015	2.96	0.1462
**X1X2**	0.0000	1	0.0000	0.0789	0.7901
**X1^2^**	0.0125	1	0.0125	25.15	0.0041
**X2^2^**	5.515 × 10^−9^	1	5.515 × 10^−9^	0.0000	0.9975
***ZP (No significant model (p > 0.05))***					
**X1**	272.67	1	272.67	4.36	0.0910
**X2**	85.92	1	85.92	1.38	0.2937
**X1X2**	343.82	1	343.82	5.50	0.0659
**X1^2^**	187.54	1	187.54	3.00	0.1437
**X2^2^**	51.66	1	51.66	0.8269	0.4049

X1 and X2 represent BSA concentration (*w*/*v*%) and Glutaraldehyde amount (µL). df indicates the degree of freedom.

**Table 4:** Summary of statistical parameters used to evaluate the adequacy and predictive performance of the developed models, including coefficient of determination (R^2^), adjusted R^2^, predicted R^2^, model F-values, *p*-values, and lack-of-fit test results for each response.

Response	Model	R^2^	Adjusted R^2^	Predicted R^2^	Model F-Value	*p*-Value	Lack-of-Fit (*p*-Value)
PS	No significant model	—	—	—	—	>0.05	—
PDI	Quadratic	0.9710	0.9420	0.8820	33.49	0.0008	0.7710
ZP	No significant model	—	—	—	—	>0.05	—

**Table 5:** The predicted and observed responses of the selected BSA nanoparticles based on desirability function analysis.

X1:X2	Dependent Variable	Predicted Value	Experimental Value	Error of Prediction (%)
**2.98:158.4**	Y1 (nm)	116.48	115.60	−0.75
	Y2 (PDI)	0.039	0.028	−28.2
	Y3 (mV)	−30.0	−21.8	+27.3

Note: The predicted values were obtained from the desirability function analysis and are presented for comparison purposes only, as no statistically significant models were obtained for all responses.

**Table 6:** Results for the optimized loaded and unloaded formulations.

	PS (nm ± SD)	PDI (Mean ± SD)	ZP (mV ± SD)	EE of PAZ (% ± SD)	EE of ENZ (% ± SD)
**Free-BSA**	115.6 ± 3.4	0.028 ± 0.02	−21.8 ± 1.2	-	-
**P-BSA**	141.4 ± 6.0	0.038 ± 0.05	−27.2 ± 1.8	97.82 ±1.61	-
**E-BSA**	124.05 ± 3.4	0.033 ± 0.03	−32.85 ± 1.0	-	70.53 ± 0.01
**PE-BSA**	128.7 ± 2.6	0.026 ± 0.01	−31.65 ± 1.1	98.59 ±1.78	69.79 ± 0.02

**Table 7:** Kinetic model fitting parameters for PAZ and ENZ release from PE-BSA nanoparticles.

Released Active Substance	Zero Order		First Order		Higuchi Model		Hixson–Crowell		Korsmeyer–Peppas		
	k	*r* ^2^	k	*r* ^2^	k	*r* ^2^	k	*r* ^2^	k	*n*	*r* ^2^
**PAZ**	1.17	0.4882	−0.0008	0.5029	1.5552	0.7347	0.0029	0.4973	2.3751	0.3757	0.9578
**ENZ**	1.50	0.8647	−0.0157	0.9491	11.441	0.9605	0.0405	0.9402	2.8235	0.4508	0.9269

**Table 8:** Stability results for PE-BSA after 6 months.

Time	0th Day	6th Month
**PS (nm ± SD)**	128.7 ± 2.6	133.2 ± 0.5
**PDI (mean ± SD)**	0.026 ± 0.01	0.06 ± 0.01
**ZP (Mv ± SD)**	−31.65 ± 1.13	−4.67 ± 1.14

**Table 9:** Stability results of PE-BSA in serum for 24 h.

Time (h)	0th	24th
**PS (nm ± SD)**	144.6 ± 0.3	151.3 ± 0.1
**PDI ± SD**	0.15 ± 0.02	0.070 ± 0.001

## Data Availability

The original contributions presented in this study are included in the article/Supplementary Material. Further inquiries can be directed to the corresponding author.

## Supplementary Materials

The following supporting information can be downloaded at: https://www.mdpi.com/article/10.3390/pharmaceutics18040475/s1, Figure S1. The molecular structures of (a) PAZ and (b) ENZ. Figure S2. DLS size distribution profiles of the nanoparticle formulations (Free-BSA, P-BSA, E-BSA and PE-BSA) measured by dynamic light scattering. Figure S3. Release kinetic model fitting plots for PAZ and ENZ from PE-BSA nanoparticles (Zero-order, First-order, Higuchi, Hixson–Crowell and Korsmeyer–Peppas models). (a) For PAZ; (b) For ENZ.

## References

[1] Maqbool M., Bekele F., Fekadu G. (2022). Treatment Strategies Against Triple-Negative Breast Cancer: An Updated Review. Breast Cancer Targets Ther..

[2] Obidiro O., Battogtokh G., Akala E.O. (2023). Triple Negative Breast Cancer Treatment Options and Limitations: Future Outlook. Pharmaceutics.

[3] Ye F., Dewanjee S., Li Y., Jha N.K., Chen Z.S., Kumar A., Vishakha, Behl T., Jha S.K., Tang H. (2023). Advancements in clinical aspects of targeted therapy and immunotherapy in breast cancer. Mol. Cancer.

[4] Zanardi E., Bregni G., de Braud F., Di Cosimo S. (2015). Better Together: Targeted Combination Therapies in Breast Cancer. Semin. Oncol..

[5] Funmilola A.F., Emmanuel O.A. (2019). Drug Combinations in Breast Cancer Therapy. Pharm. Nanotechnol..

[6] Sonpavde G., Hutson T.E. (2007). Pazopanib: A novel multitargeted tyrosine kinase inhibitor. Curr. Oncol. Rep..

[7] Sloan B., Scheinfeld N.S. (2008). Pazopanib, a VEGF receptor tyrosine kinase inhibitor for cancer therapy. Curr. Opin. Investig. Drugs.

[8] Gril B., Palmieri D., Qian Y., Smart D., Ileva L., Liewehr D.J., Steinberg S.M., Steeg P.S. (2011). Pazopanib Reveals a Role for Tumor Cell B-Raf in the Prevention of HER2+ Breast Cancer Brain Metastasis. Clin. Cancer Res..

[9] Xu Z., Zhou Z., Yang X., Thakur A., Han N., Li H.-T., Li L.-G., Hu J., Li T.-F., Yan Y. (2024). Determining M2 macrophages content for the anti-tumor effects of metal-organic framework-encapsulated pazopanib nanoparticles in breast cancer. J. Nanobiotechnol..

[10] Giovannelli P., Di Donato M., Galasso G., Di Zazzo E., Bilancio A., Migliaccio A. (2018). The Androgen Receptor in Breast Cancer. Front. Endocrinol..

[11] Xia X., Huang C., Liao Y., Liu Y., He J., Guo Z., Jiang L., Wang X., Liu J., Huang H. (2019). Inhibition of USP14 enhances the sensitivity of breast cancer to enzalutamide. J. Exp. Clin. Cancer Res..

[12] Cochrane D.R., Bernales S., Jacobsen B.M., Cittelly D.M., Howe E.N., D’Amato N.C., Spoelstra N.S., Edgerton S.M., Jean A., Guerrero J. (2014). Role of the androgen receptor in breast cancer and preclinical analysis of enzalutamide. Breast Cancer Res..

[13] Choupani E., Madjd Z., Saraygord-Afshari N., Kiani J., Hosseini A. (2022). Combination of androgen receptor inhibitor enzalutamide with the CDK4/6 inhibitor ribociclib in triple negative breast cancer cells. PLoS ONE.

[14] Tran P., Lee S.-E., Kim D.-H., Pyo Y.-C., Park J.-S. (2020). Recent advances of nanotechnology for the delivery of anticancer drugs for breast cancer treatment. J. Pharm. Investig..

[15] Avitabile E., Bedognetti D., Ciofani G., Bianco A., Delogu L.G. (2018). How can nanotechnology help the fight against breast cancer?. Nanoscale.

[16] Elzoghby A.O., Samy W.M., Elgindy N.A. (2012). Albumin-based nanoparticles as potential controlled release drug delivery systems. J. Control. Release.

[17] Rahimnejad M., Jahanshahi M., Najafpour G. (2006). Production of biological nanoparticles from bovine serum albumin for drug delivery. Afr. J. Biotechnol..

[18] Esim O., Kiymaci M.E., Hascicek C. (2023). Ciprofloxacin HCl-loaded Albumin Nanoparticles for the Treatment of Recurrent Urinary Tract Infections: Preparation, Optimization, and Evaluation of Antibacterial Activity. J. Pharm. Innov..

[19] Sankar P.R., Latha K.S., Sailu A.B., Taheera S., Madhuri B. (2021). Development and validation of RP-HPLC method for the determination of Pazopanib Hydrochloride (A tyrosine kinase inhibitor) in pharmaceutical dosage form. Res. J. Pharm. Technol..

[20] Gungor S., Akbel E., Bulduk İ. (2023). Development, Optimization, and Validation of HPLC Method for Quantification of Enzalutamide in Bulk and Pharmaceuticals. Pharm. Chem. J..

[21] Secerli J., Adatepe Ş., Altuntas S., Topal G.R., Erdem O., Bacanlı M. (2023). In vitro toxicity of naringin and berberine alone, and encapsulated within PMMA nanoparticles. Toxicol. Vitr..

[22] Shinde A., Panchal K., Patra P., Singh S., Enakolla S., Paliwal R., Chaurasiya A. (2024). QbD Enabled Development and Evaluation of Pazopanib Loaded Nanoliposomes for PDAC Treatment. AAPS PharmSciTech.

[23] Ramana P.V., Krishna Y.R., Mouli K.C. (2022). Experimental FT-IR and UV–Vis spectroscopic studies and molecular docking analysis of anti-cancer drugs Exemestane and Pazopanib. J. Mol. Struct..

[24] Pawar A., Dhudum R., Kandhare P., Ganeshpurkar A. (2025). Development and Characterization of Enzalutamide-Loaded Solid Lipid Nanoparticles for Enhanced Drug Delivery System. Biomed. Mater. Devices.

[25] Medarametla R.T., Radha G.V. (2025). QbD Based Development, Characterization and Pharmacokinetic Evaluation of Enzalutamide-Loaded Cyclodextrin Nano Sponges for Enhanced Drug Delivery. Indian J. Pharm. Educ. Res..

[26] Bazaei M., Honarvar B., Esfandiari N., Sajadian S.A., Aboosadi Z.A. (2024). Production of pazopanib hydrochloride nanoparticles (anti-kidney cancer drug) using a supercritical gas antisolvent (GAS) method. RSC Adv..

[27] Song M.-M., Branford-White C., Nie H.-L., Zhu L.-M. (2011). Optimization of adsorption conditions of BSA on thermosensitive magnetic composite particles using response surface methodology. Colloids Surf. B Biointerfaces.

[28] Sen Gupta A. (2016). Role of particle size, shape, and stiffness in design of intravascular drug delivery systems: Insights from computations, experiments, and nature. Wiley Interdiscip. Rev. Nanomed. Nanobiotechnol..

[29] Chu Y., Chai S., Pan H., Qian J., Han C., Sui X., Liu T. (2022). Halogenated Salts as Coagulant to Prepare Bovine Serum Albumin Nanoparticles Containing Paclitaxel using High-Pressure Homogenisation Method. Indian J. Pharm. Sci..

[30] Srivastava A. (2019). Preparation, Characterization and Encapsulation Efficiency of Egg Albumin Nanoparticles Using EDC as Crosslinker. J. Sci. Ind. Res..

[31] Bartlett B.A., Klier J., Razavi S. (2025). Preparation of bovine serum albumin nanospheres via desolvation: A study of synthesis, characterization, and aging. Nanoscale.

[32] Danaei M., Dehghankhold M., Ataei S., Hasanzadeh Davarani F., Javanmard R., Dokhani A., Khorasani S., Mozafari M.R. (2018). Impact of Particle Size and Polydispersity Index on the Clinical Applications of Lipidic Nanocarrier Systems. Pharmaceutics.

[33] Fadilah F., Febriani E., Kurniasari E., Nurfikhri R., Sirait T. (2023). Encapsulation of Rice Bran Oil (RBO) by Complex Coacervation Using Glutaraldehyde as Crosslinking Agent. Equilib. J. Chem. Eng..

[34] Dubey R.D., Alam N., Saneja A., Khare V., Kumar A., Vaidh S., Mahajan G., Sharma P.R., Singh S.K., Mondhe D.M. (2015). Development and evaluation of folate functionalized albumin nanoparticles for targeted delivery of gemcitabine. Int. J. Pharm..

[35] Attia M.S., Radwan M.F., Ibrahim T.S., Ibrahim T.M. (2023). Development of Carvedilol-Loaded Albumin-Based Nanoparticles with Factorial Design to Optimize In Vitro and In Vivo Performance. Pharmaceutics.

[36] Bhatia M., Devi R. (2019). Enhanced solubility and drug release of ketoprofen using lyophilized bovine serum albumin solid dispersion. ACTA Pharm. Sci..

[37] Khoder M., Abdelkader H., ElShaer A., Karam A., Najlah M., Alany R.G. (2016). Efficient approach to enhance drug solubility by particle engineering of bovine serum albumin. Int. J. Pharm..

[38] Dawoud M.H.S., Abdel-Daim A., Nour M.S., Sweed N.M. (2023). A Quality by Design Paradigm for Albumin-Based Nanoparticles: Formulation Optimization and Enhancement of the Antitumor Activity. J. Pharm. Innov..

[39] Baishya H. (2017). Application of Mathematical Models in Drug Release Kinetics of Carbidopa and Levodopa ER Tablets. J. Dev. Drugs.

[40] Choi J.-S., Meghani N. (2016). Impact of surface modification in BSA nanoparticles for uptake in cancer cells. Colloids Surf. B Biointerfaces.

[41] Pooja D., Panyaram S., Kulhari H., Rachamalla S.S., Sistla R. (2014). Xanthan gum stabilized gold nanoparticles: Characterization, biocompatibility, stability and cytotoxicity. Carbohydr. Polym..

[42] Teixeira S., Ferreira D., Rodrigues L.R., Carvalho M.A., Castanheira E.M.S. (2025). Albumin/Hyaluronic Acid Gel Nanoparticles Loaded with a Pyrimidine-Based Drug for Potent Anticancer Activity. Gels.

[43] Akanda M., Getti G., Douroumis D. (2023). In vivo evaluation of nanostructured lipid carrier systems (NLCs) in mice bearing prostate cancer tumours. Drug Deliv. Transl. Res..

[44] Guo Q., Wang S., Xu R., Tang Y., Xia X. (2024). Cancer cell membrane-coated nanoparticles: A promising anti-tumor bionic platform. RSC Adv..

[45] Ferreira L.F., Picco A.S., Galdino F.E., Albuquerque L.J.C., Berret J.F., Cardoso M.B. (2022). Nanoparticle-Protein Interaction: Demystifying the Correlation between Protein Corona and Aggregation Phenomena. ACS Appl. Mater. Interfaces.

[46] Cao Y., Wang B., Wang Y., Lou D. (2014). Dual Drug Release from Core–Shell Nanoparticles with Distinct Release Profiles. J. Pharm. Sci..

[47] Hemlata, Hariharan A., Murali N., Roy S., Betal S., Kumar S., Minocha S. (2025). Engineered BSA nanoparticles: Synthesis, drug loading, and advanced characterization. Biol. Methods Protoc..

[48] Lu Y.-L., Ma Y.-B., Feng C., Zhu D.-L., Liu J., Chen L., Liang S.-J., Dong C.-Y. (2019). Co-delivery of Cyclopamine and Doxorubicin Mediated by Bovine Serum Albumin Nanoparticles Reverses Doxorubicin Resistance in Breast Cancer by Down-regulating P-glycoprotein Expression. J. Cancer.

[49] Tiruppathi C., Song W., Bergenfeldt M., Sass P., Malik A.B. (1997). Gp60 Activation Mediates Albumin Transcytosis in Endothelial Cells by Tyrosine Kinase-dependent Pathway. J. Biol. Chem..

[50] Esim O., Sarper M., Ozkan C.K., Oren S., Baykal B., Savaser A., Ozkan Y. (2020). Effect simultaneous delivery with P-glycoprotein inhibitor and nanoparticle administration of doxorubicin on cellular uptake and in vitro anticancer activity. Saudi Pharm. J..

[51] Güney G., Kutlu H.M., Genç L. (2014). Preparation and characterization of ascorbic acid loaded solid lipid nanoparticles and investigation of their apoptotic effects. Colloids Surf. B Biointerfaces.

[52] Topal G.R., Adatepe S., Secerli J., Gudul Bacanli M. (2025). Development and estimation of lipid hybrid nanocarriers co-loaded oxaliplatin and melatonin for breast cancer. Drug Dev. Ind. Pharm..

