# Supplementary material for: BSA-Based Nanoparticles for Dual Loading of Pazopanib and Enzalutamide: Formulation Optimization and In Vitro Evaluation in Breast Cancer Cells
Source: Pharmaceutics. 2026 Apr 13;18(4):475. doi: 10.3390/pharmaceutics18040475 (PMC13119014; doi:10.3390/pharmaceutics18040475)
Supplement: Supplementary file 1 [file pharmaceutics-18-00475-s001.zip › pharmaceutics-4223342-supplementary.pdf]

Supplemantary Data

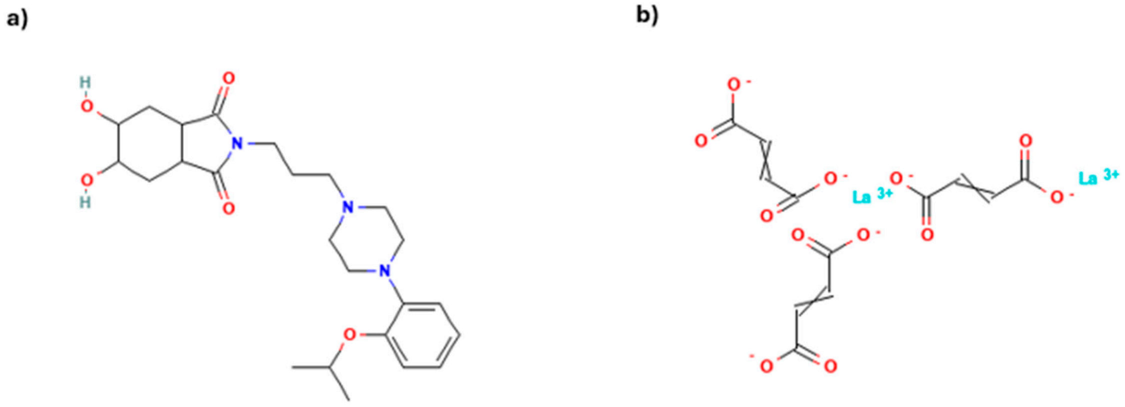

Figure S1. The molecular structures of a) PAZ and b) ENZ

a) **Free BSA**

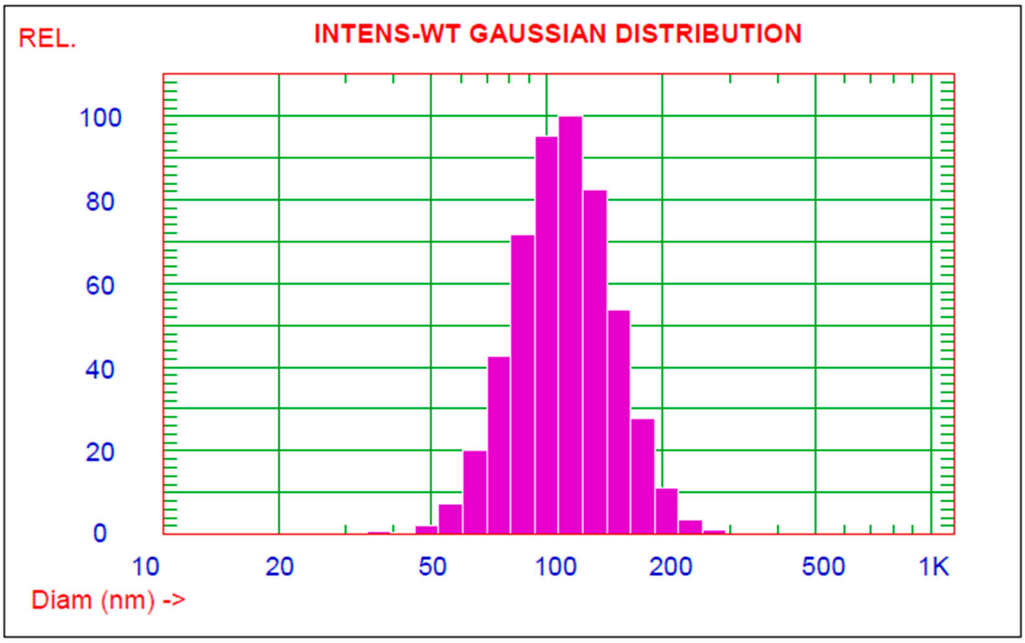

b) **P-BSA**

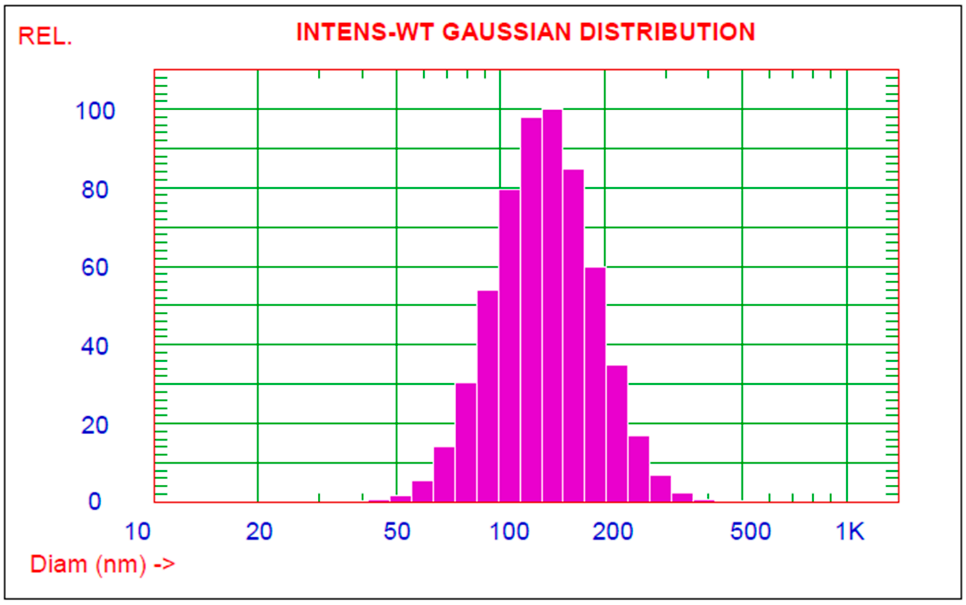

c) E-BSA

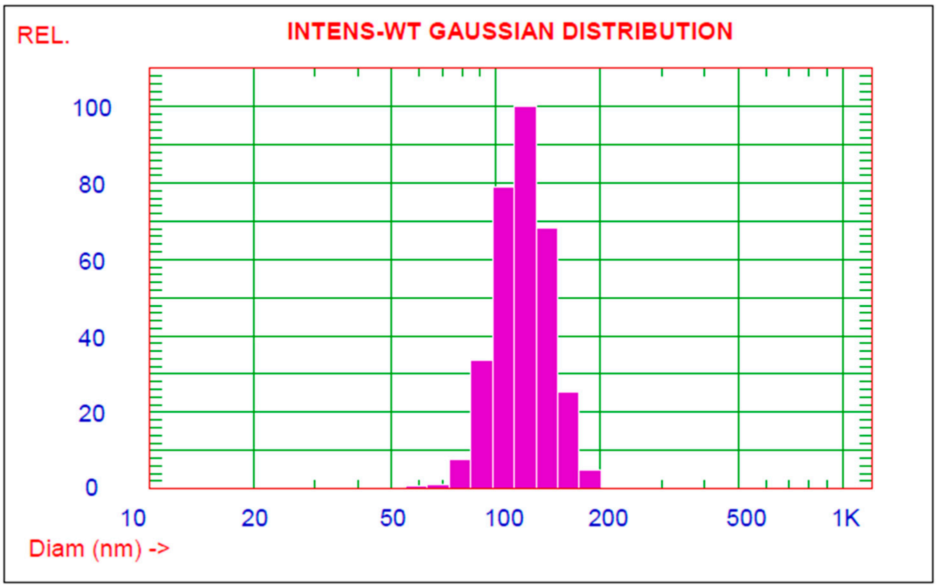

d) PE-BSA

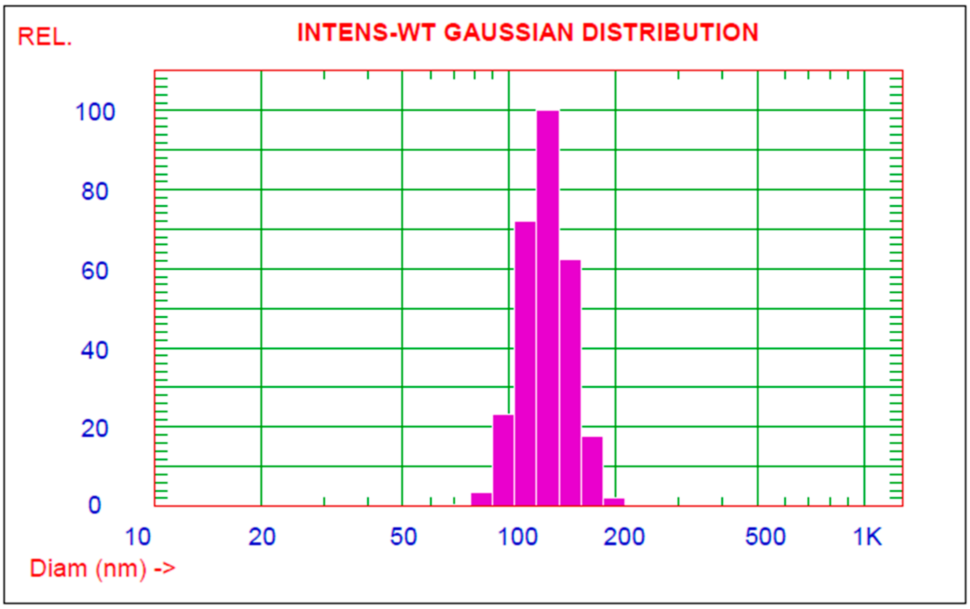

Figure S2. DLS size distribution profiles of the nanoparticle formulations (Free-BSA, P-BSA, E-BSA and PE-BSA) measured by dynamic light scattering.

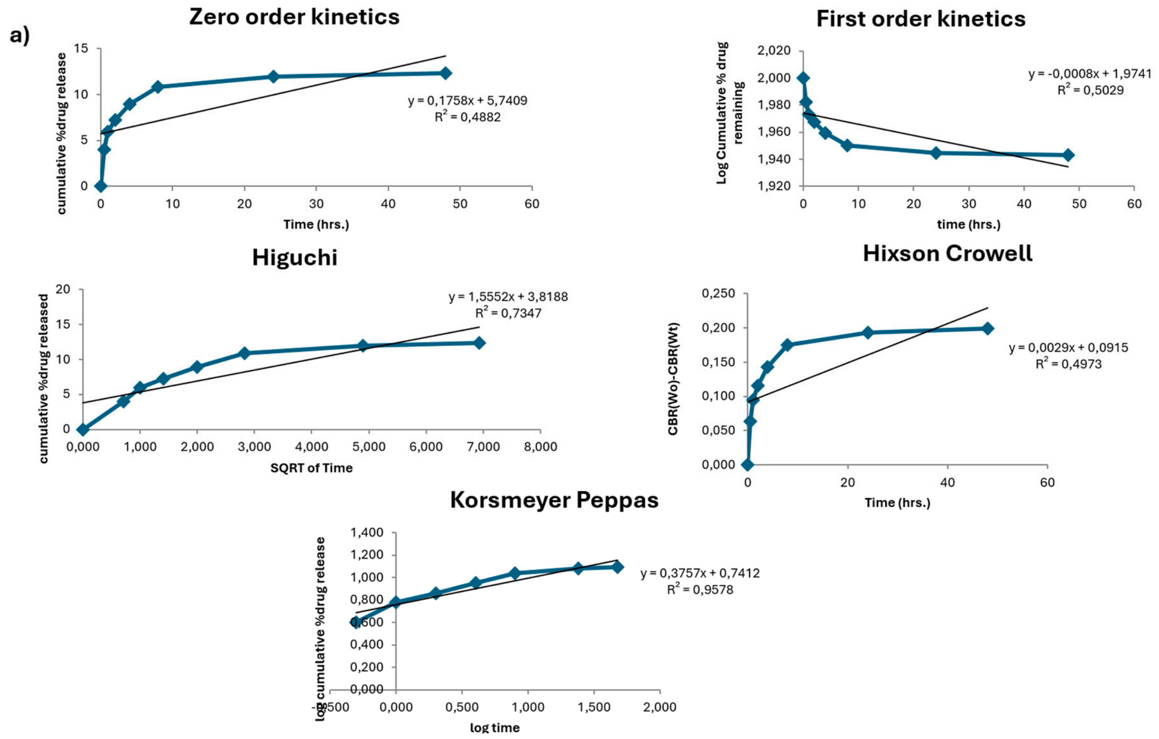

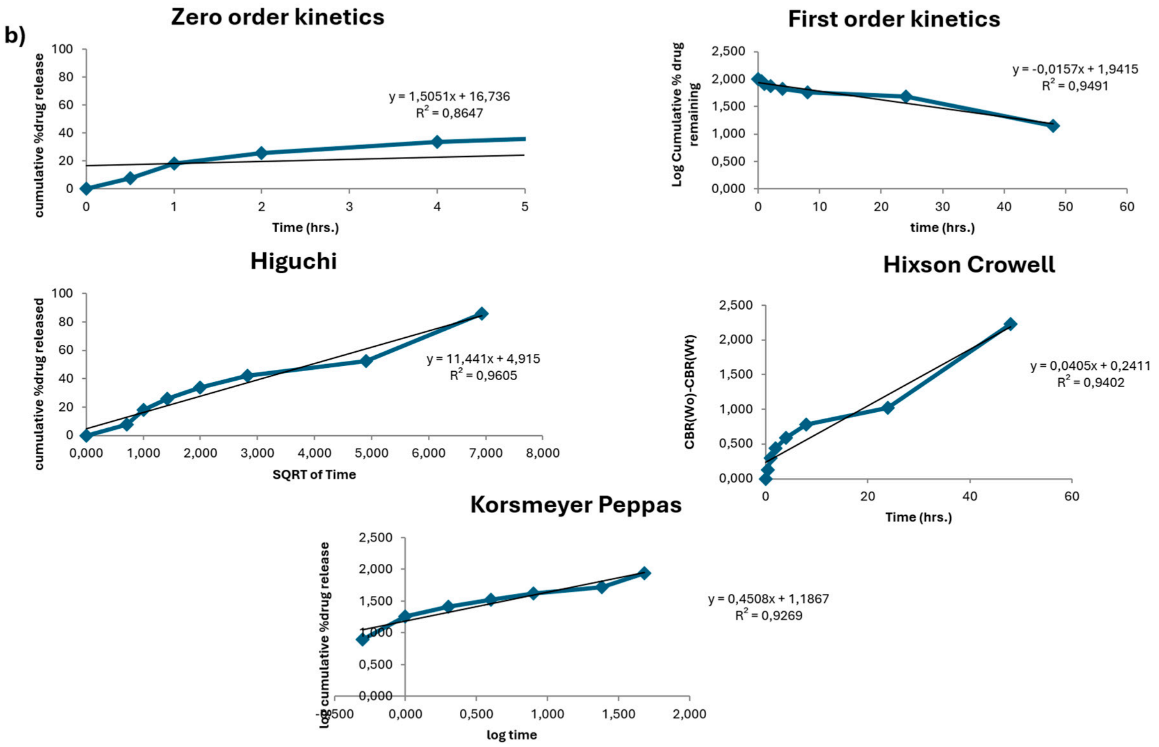

Figure S3. Release kinetic model fitting plots for PAZ and ENZ from PE-BSA nanoparticles (Zero-order, First-order, Higuchi, Hixson–Crowell and Korsmeyer–Peppas models). a) For PAZ; b) For ENZ
